# In vitro modulation of mTOR and mGlur5 influence α-synuclein accumulation

**DOI:** 10.1186/s13041-023-01074-2

**Published:** 2024-02-15

**Authors:** Viktoria Xing, Kyle Biggar, Stephen S. G. Ferguson, Shawn Hayley

**Affiliations:** 1 Department of Neuroscience, Carleton University, 1125 Colonel By Drive, Ottawa, ON K1S 5B6 Canada; 2 Institute of Biochemistry and Department of Biology, Carleton University, 1125 Colonel By Drive, Ottawa, ON K1S 5B6 Canada; 3 Department of Cellular and Molecular Medicine, Faculty of Medicine, University of Ottawa Brain and Mind Research Institute, University of Ottawa, 451 Smyth Road, Ottawa, ON K1H 8M5 Canada

**Keywords:** α-synuclein, mGluR5, Parkinson’s disease, mTOR, In vitro

## Abstract

One of the main hallmarks of Parkinson’s disease (PD) is abnormal alpha-synuclein (α-syn) aggregation which forms the main component of intracellular Lewy body inclusions. This short report used preformed α-syn fibrils, as well as an A53T mutant α-syn adenovirus to mimic conditions of pathological protein aggregation in dopaminergic human derived SH-SY5Y neural cells. Since there is evidence that the mTOR pathway and glutamatergic signaling each influence protein aggregation, we also assessed the impact of the mTOR inhibitor, rapamycin and the mGluR5 allosteric modulator, CTEP. We found that both rapamycin and CTEP induced a significant reduction of α-syn fibrils in SH-SY5Y cells and this effect was associated with a reduction in mTOR signaling and enhancement in autophagic pathway factors. These data support the possibility that CTEP (or rapamycin) might be a useful pharmacological approach to target abnormal α-syn accumulation by promoting intracellular degradation or enhanced clearance.

## Introduction

Parkinson’s disease (PD) is associated with the accumulation of pathological proteinaceous inclusions, referred to as Lewy bodies [4, 9, 34, 36, 38]. A substantial component of Lewy bodies comprises abnormal misfolded α-synuclein (α-syn) protein, which can become pathological when adopting oligomeric or fibril forms [17], and appears to be able to spread from cell to cell in a prion-like fashion [34]. Indeed, α-synuclein is normally a presynaptic cytosolic monomer but can undergo oligomerization and fibrillation contributing to synucleopathies [5, 14, 39]. Competing theories suggest that α-syn may first begin to misfold and aggregate in either the periphery and/or in the anterior olfactory region of the brain before eventually reaching the midbrain dopaminergic system [6, 7, 48]. Aggregation and spread of α-synuclein can be affected by several missense mutations, including A30P, E46K, H50Q and A53T [8], with the A53T mutation being particularly involved in the acceleration of fibrilization [41, 42].

At least part of the problematic accumulation of pathological α-synuclein protein likely involves deficits in the cellular processes that are responsible for the degradation of misfolded proteins. In particular, autophagy, which is mediated by the mammalian target of rapamycin (mTOR) pathway, is critical for preventing excessive protein aggregation [22]. Normally, mTORC1 has an inhibitory role on downstream initiators of autophagy and consequently, blocking this inhibitory process can augment the autophagic removal of toxic proteins. In fact, mTORC1 inhibition, using the antagonist, rapamycin, does indeed increase autophagy [28] and was protective against the dopaminergic neurotoxins, rotenone and 6-OHDA [18, 29].

The Gα protein-coupled metabotropic glutamate receptor 5 (mGluR5) has also been found to modulate autophagy and may do so through regulation of mTOR signaling [1, 37]. There are several mGluR5 acting pharmacological agents, but the negative allosteric modulator, CTEP (2-chloro-4-[2-[2,5-dimethyl-1-[4 (trifluoromethoxy)phenyl]imidazole 4yl]ethynyl] pyridine), is of particular interest given its favorable pharmacokinetic profile [24] and the fact that CTEP prevented huntingtin and β-amyloid aggregates in animal models of the Huntington’s and Alzheimer’s disease through the regulation of mTOR and autophagy [1–3].

Many recent studies have utilized administration of the exogenous preformed-fibril form of α-syn (PFFs) to promote in vivo and in vitro α-syn aggregation and toxicity [5, 43, 44, 46]. We presently sought to assess the impact of CTEP in comparison to the direct mTOR inhibitor, rapamycin, upon α-syn levels within PFF treated human dopaminergic SH-SY5Y cells. To this end, CTEP and rapamycin reduced α-syn fibril levels in SH-SY5Y cells that had accumulated the PFFs. This reduced α-syn burden was associated with signs of enhanced mTOR activity and autophagic proteins. These data provide a novel in vitro approach to model α-syn fibril accumulation and suggest the importance of mTOR in this process.

## Methods

### Model development

SH-SY5Y, human neuroblastoma, cells were selected for their dopamine neuron-like qualities and their robust survivability and steady growth. Cell culture was maintained by weekly passage. Preformed α-syn fibrils were prepared from α-syn monomer (Proteos #R003) and added to experimental culture to mimic pathological α-syn aggregation. The optimal concentration of α-syn fibrils was determined through assessment of immunofluorescence-stained SH-SY5Y cells treated with α-syn fibrils at 1:100, 1:200, 1:400, 1:800 and 1:1000 ratios from stock solution of 5 mg/ml. Next, a CTEP dose–response was determined through assessment of SH-SY5Y cells treated with α-syn fibrils at a ratio of 1:100. Specifically, CTEP was applied to cells at 0.1 μM, 1 μM, 10 μM and 100 μM concentrations. A rapamycin concentration of 5 μM was selected for use based on existing reports [23] and on our own recent experiments.

### Cell culture procedures

SH-SY5Y cells were plated at 10^4^ cells/mL to 6-well plates for Western Blot, and at 5 × 10^3^ cells/mL to 96-well plate for immunostaining. Cell growth media contained 10% of FBS and 1% of Pen-Strep (stock concentration: 10,000–12000 units/mL). Cells were differentiated with three treatments of retinoic acid (10 μM). After a further 48 h, α-syn fibrils (1:100; determined to be optimal) and A53T AV (1 MOI) (or vehicle) were added and cells were incubated for 6 days. On the 6th day, vehicle, CTEP (10 μM) or rapamycin (5 μM) were added to their respective treatment groups. After 24 h incubation with vehicle, CTEP or rapamycin, cells were either harvested and preserved in -80 °C for western blot analyses, or fixed with 4% PFA for immunofluorescence. All cell culture experiments were conducted on three different times to yield an n = 3 for all treatment groups.

### Immunofluorescence analyses

SH-SY5Y cells were first fixed with 4% PFA for 15 min. Following three PBS washes, the cells were then blocked in 2% BSA with 0.1% Triton-X diluted in PBS for 30 min. Thereafter, cells were incubated with primary rabbit α-syn antibody (conformation specific antibody that primarily labels the α-syn “filament” or aggregate form of the protein; MJFR-14–6-4–2, ab209538, 1:1000) in 0.1% BSA solution for 1 h and following further PBS washes, they were incubated with secondary goat anti-rabbit Alexa Fluor 488 antibody in 0.1% BSA solution for 30 min. Finally, cells were washed three times in PBS and imaged with ThermoFisher EVOS FL Auto 2 system (at × 40 magnification). The labeled α-syn fibrils were counted using an Image-J software particle counting program. Briefly, images of the same magnification and background were imported into Image-J and scale parameters were set. An identical region of interest was demarcated for each image which included the particles of interest. Next, a set threshold was established and applied to all images in order to minimize background fluorescence and capture positive particles.

### Western Blot assays

Protein from SH-SY5Y cells preserved at -80 °C was extracted using RIPA buffer (0.1% SDS, 1 mM Na ortho-vandate in 10 mM tris) with addition of EDTA-free protease inhibitors (Roche cOmplete). Samples were sonicated in the presence of the extraction buffer for 5 cycles, 30 s each and then protein content was determined by Pierce’s BCA Protein Assay Kit. Samples were diluted with loading buffer and loaded into acrylamide mini-gels of appropriate concentration and run at 140 V. Then gels were transferred onto MeOH activated PVDF membranes at 100 V. Membranes were dried overnight and reverted with MeOH. To acquire normalized signal, membranes were stained with FastGreen. Membranes were then blocked with 0.5% fish gelatin for 30 min, and then incubated with primary antibody [mTOR (CAT#2983); p-mTOR Ser2448 (CAT5536); AKT (CAT#9272); p-AKT Ser473 (CAT#4060); p-p70S Thr389 (CAT#9205) from Cell Signaling Tech] at 1:000 in 0.05% fish gelatin solution for 2 h. After 4 TBS-T washes, membranes were incubated with secondary antibody in 0.5% fish gelatin solution for 1 h. After 4xTBS-T 5 min and 2xTBS 5 min washes membranes were finally imaged with Licor Odyssey FC imaging system. Images were further quantified with Image Studio Lite 5.2 software.

### Statistics

Using a Prism statistical software package (Version 9.3), three-way 2 (Non-fibril vs Fibril) × 2 (No AS53T AV vs A53T AV) × 3 (Vehicle vs CTEP vs Rapamycin) ANOVAs were conducted as appropriate. A biological n = 3 (Western blot) or 9 (Immunofluorescence) (with multiple technical replicates for each) was used for all statistical analyses. Follow up post-hoc testing was completed using Tukey’s pairwise comparisons (p < 0.05).

## Results

**Fig. 1:**
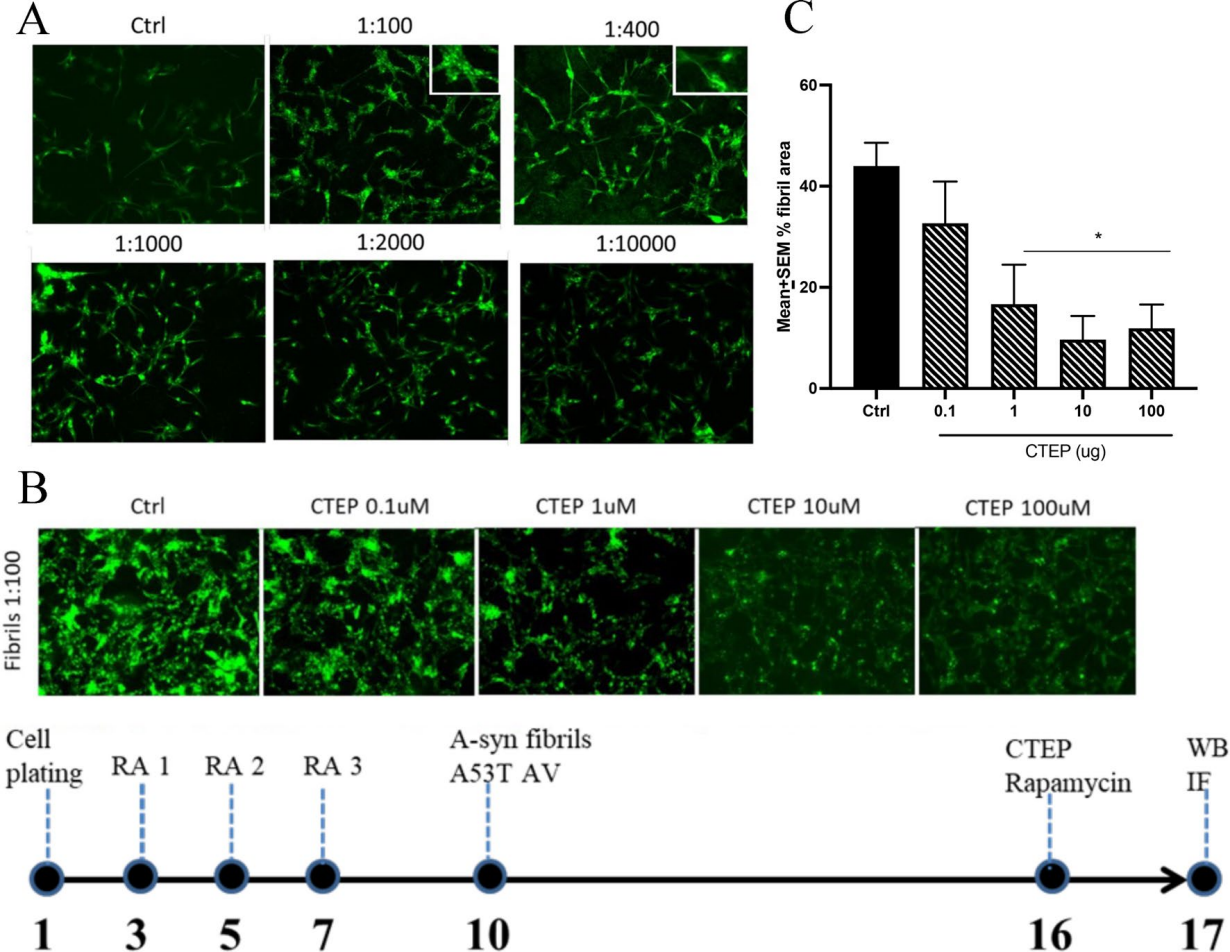
Experimental timeline (bottom). On Days 3, 5 and 7 after plating the SH-SY5Y cells were treated with retinoic acid (RA). Then α-syn fibrils and/or A53T AV were added on Day 10. After a further 6 days, treatment with CTEP or rapamycin was applied on Day 16. SH-SY5Y cells were then harvested on Day 17 after after a final 24 h. **A.** Testing a series of α-syn PFF concentrations revealed an optimal dilution of 1:100. As shown in the inset, this concentration produced robust punctate-like intracellular inclusions and was used for subsequent experiments. **B.** CTEP dose-dependently reduced α-syn accumulation in SH-SY5Y that were previously incubated with α-syn fibrils (1:100) for 6 days. The cells were incubated with media containing CTEP for a further 24 h and a concentration of 10 μM was found to be optimal and adopted for further experiments. **C.** The bar graph shows the quantification of α-syn labelling as a function of CTEP concentration applied

We first determined the working concentration of α-syn fibrils to be added to the SH-SY5Y cells. The α-syn concentration of 1:100 was determined to be optimal to induce sufficient, but not overwhelming cellular labelling. While the actual overall immunofluorescent intensity did not vary greatly between 1:100 to 1:400, the 1:100 concentration produced α-syn labelling that was clearly more punctate with apparent intracellular fibrous inclusions (Fig. 1A, inset). We initially conducted a small dose–response experiment to determine if CTEP actually influences α-syn fibril aggregation and if so, what is the optimal concentration? To this end, we found that CTEP clearly did dose-dependently reduce α-syn labelling [F(4,10) = 5.65, p < 0.05] and it appeared that the optimal range was between 10 μM to 100 μM (Fig. 1B). Hence, we opted to use 10 μM of CTEP in the ensuing experiment.

**Fig. 2:**
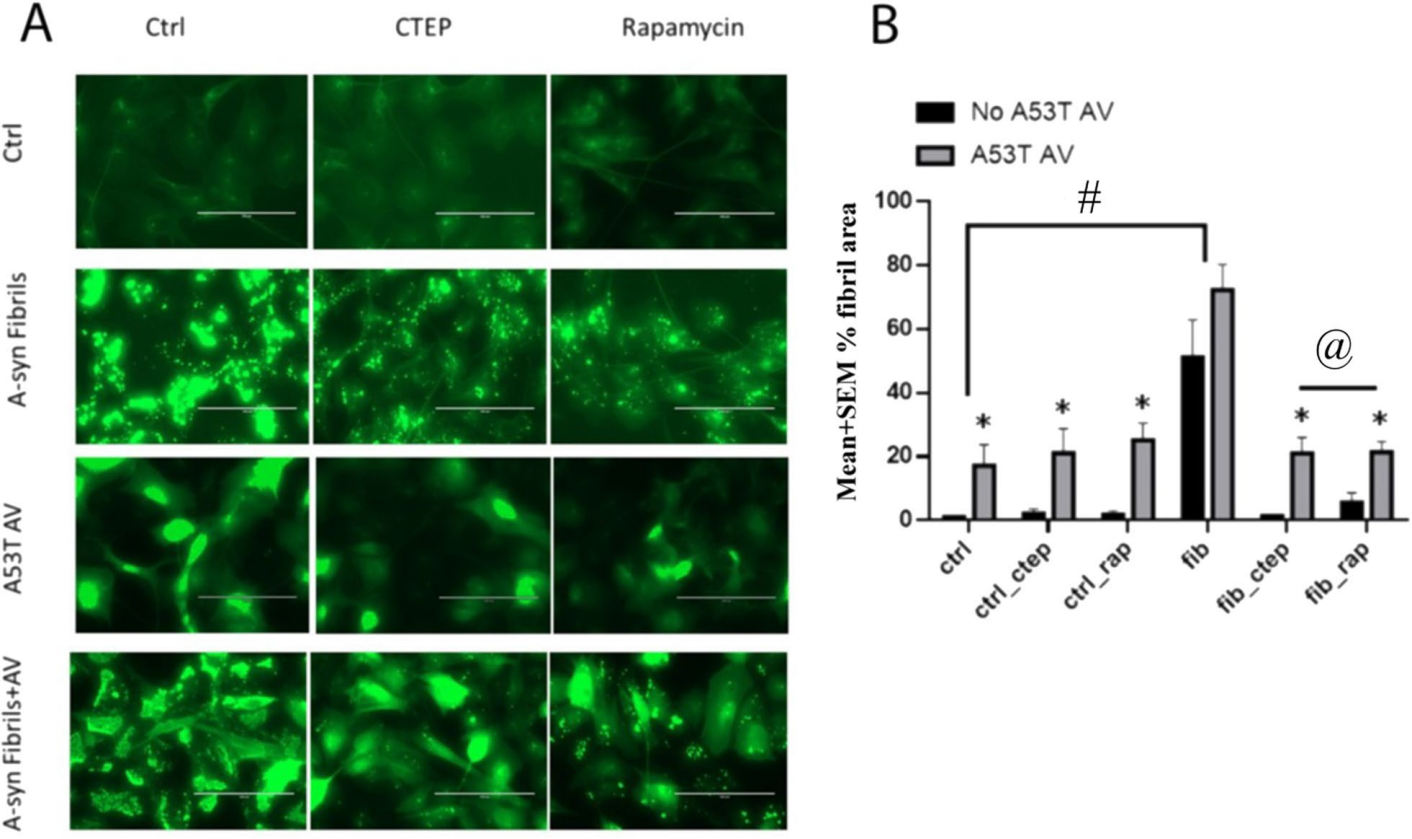
**A.** The impact of 24 h of CTEP or rapamycin exposure upon α-syn aggregation in SH-SY5Y cells previously exposed to α-syn PFFs (fib) for a six-day period. A significant reduction of α-syn positive fibril particle area was observed with both the CTEP and rapamycin (rap) treatments in response to the α-syn fibrils alone or in combination with the A53T AV. The A53T AV alone increased overall cellular fluorescence (in the absence of obvious intracellular particles) and this was also reduced by CTEP and rapamycin. **B.** Quantified data were derived from separate experiments (n = 9/group) with each carried out in triplicate. (* p < 0.05, difference compared to no A53T AV; # p < 0.05, difference compared to vehicle controls (ctrl); @ p < 0.05, difference compared to α-syn PFF (fib) treatment in the absence of CTEP or rap). Particle count was performed with Image J and statistical analysis: SPSS. Tukey’s pairwise comparison was for post hoc comparisons. Scale bars: 100 μm

While the three way ANOVA interaction just failed to meet significance F(2,96) = 2.91; p = 0.059, the immunofluorescent α-syn particle accumulation did vary as a function of a (No fibril vs fibril) x (Vehicle vs CTEP vs Rapamycin) interaction, F(2,96) = 256.6; p < 0.0001. As shown in Fig. 2, the follow up comparisons revealed that the PFF fibril treatment alone significantly increased α-syn accumulation, but that this effect was reversed when cells were also treated with either CTEP or rapamycin (p < 0.05). There was also the presence of a main effect (but no interaction with the other variables) for the A53T AV treatment, F(1,96) = 315.4; p < 0.0001. Indeed, the A53T AV treatment increased overall α-syn accumulation, compared to cells cultured in the absence of the adenoviral induced expression (p < 0.001; Fig. 2).

**Fig. 3:**
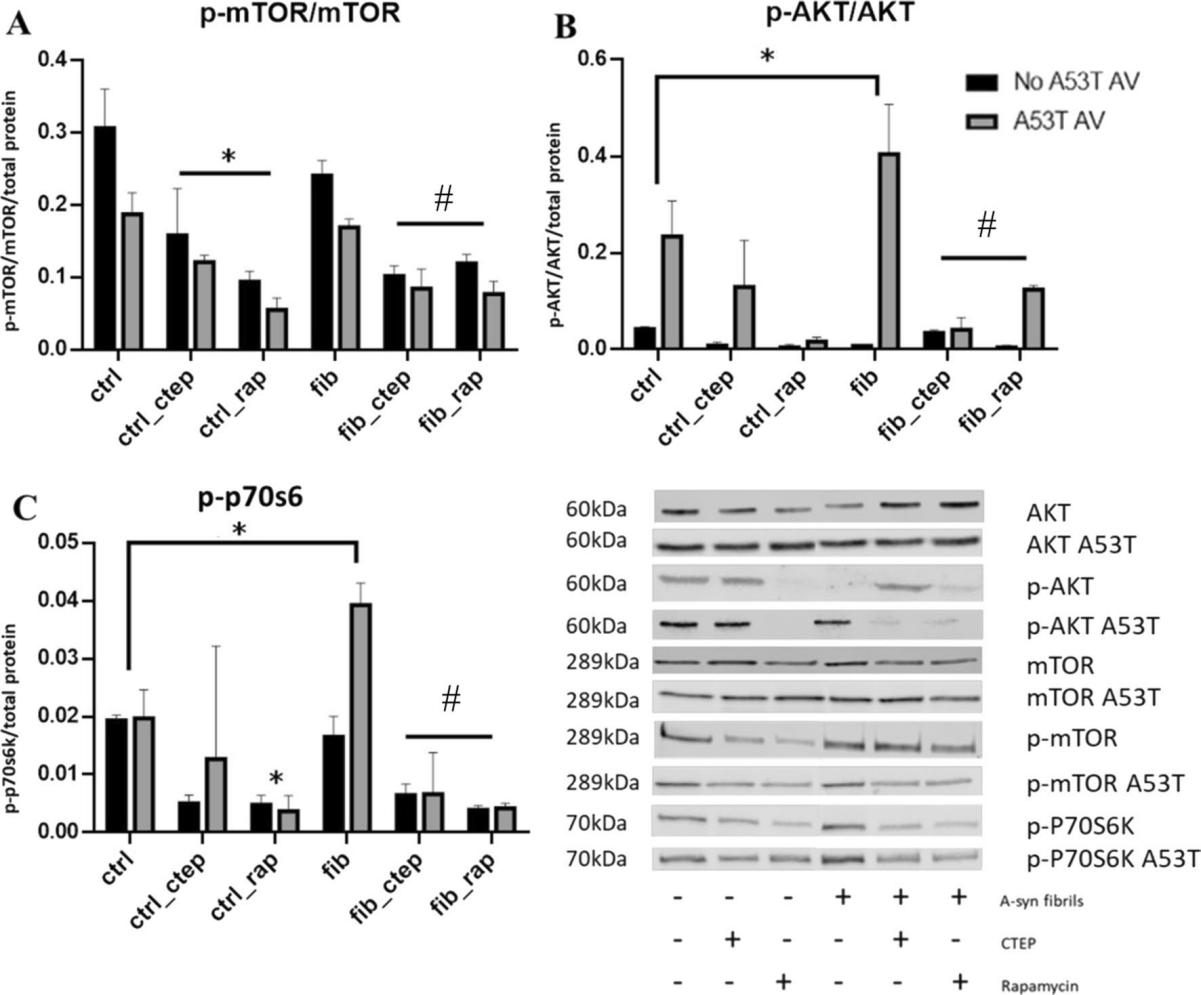
The mTOR pathway was affected by CTEP and rapamycin treatments alone and in the context of α-syn fibril exposure. **A.** The ratio of phosphorylated, p-mTOR, to total, mTOR, was taken as an index of mTOR activation. The CTEP and rapamycin (rap) treatments both reduced p-mTOR/mTOR protein levels, relative to either vehicle treated controls (ctrl) or treatment with α-syn fibril (fib) ± A53T AV exposure. **B.** The α-syn fibril + A53T AV combination treatment increased p-AKT/AKT levels above controls and this was reduced by both CTEP and rapamycin treatments. **C.** The α-syn fibril + A53T AV treatment combination increased p-p70s6 protein levels, relative to vehicle treated controls and this was markedly reduced by both the CTEP and rapamycin treatments. (* p < 0.05, difference compared to vehicle control (ctrl); # p < 0.05, difference compared α-syn fibril (fib) alone treatment). Presented data are the result of three separate experiments (n = 3/group) with each carried out in triplicate. All blot signals were normalized to total protein content. Following significant ANOVAs, Tukey’s pairwise comparison was used for post hoc comparisons

The protein levels of p-mTOR/mTOR were found to vary as a function of a (Vehicle vs CTEP vs Rapamycin) x (No Fibril vs Fibril) interaction, F(2,24) = 5.35; p < 0.05. Specifically, both CTEP and rapamycin reduced p-mTOR/mTOR levels, relative to either vehicle (ctrl) or PFF (fib) only treated groups (p < 0.05). Moreover, rapamycin also diminished levels further still in relation to CTEP treated control cells (i.e. those that did not receive the PFF fibrils) (p < 0.05; Fig. 3). There was also a significant (No A53T AV vs A53T AV) x (Vehicle vs CTEP vs Rapamycin) interaction, F(2,24) = 4.49; p < 0.05. In this case, it was found that the A53T AV reduced p-mTOR/mTOR levels, relative to the no A53T AV treatment group (p < 0.05); but no such difference between A53T AV groups was evident for CTEP or rapamycin treated cells.

The levels of pAKT/AKT varied as a complex significant three way (No fibril vs Fibril) x (No A53T AV vs A53T AV) x (Vehicle vs CTEP vs Rapamycin) interaction, F(2,24) = 14.11; p < 0.001. The follow up comparisons revealed that the A53T AV alone increased pAKT/AKT levels and that this was further augmented with the addition of the fibrils, relative to control cells (p < 0.05). As evident in Fig. 3, the CTEP and rapamycin treatments both reversed the impact of the combined A53T AV + fibril exposure (p < 0.05).

The levels of p-p70S6 varied as a function of a (Vehicle vs CTEP vs Rapamycin) x (No fibril vs Fibril) x (No A53T AV vs A53T AV) interaction, F(2,24) = 4.21; p < 0.05. Further comparisons between the groups revealed that the combined (but not individual) PFF fibril and A53T AV treatments increased p-p70S6 levels compared to vehicle treatment (p < 0.05). Once again, as shown in Fig. 3, the CTEP and rapamycin treatments greatly reduced this effect to a level even below that of vehicle treated control cells (p < 0.05).

**Fig. 4:**
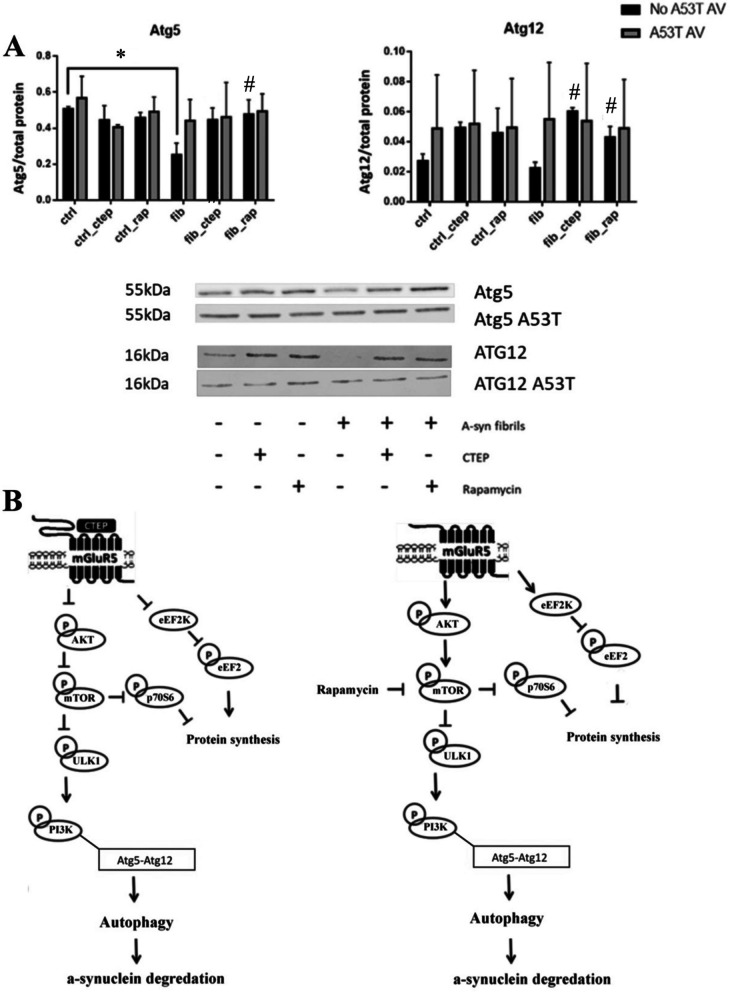
The Atg5 and Atg12 autophagy linked proteins were affected by CTEP and rapamycin treatments in the context of exogenous α-syn fibril exposure (black bars). **A.** Atg5 levels were significantly reduced by exogenously applied α-syn preformed fibrils (fib). The CTEP and rapamycin (rap) treatments reversed this α-syn fibril Atg5 reduction, as well as increasing Atg12 levels in the context of the α-syn PFFs. The A53T AV treated cells did not differ whatsoever between the groups (grey bars). (* p < 0.05, difference compared to vehicle control (ctrl); # p < 0.05, difference compared α-syn fibril (fib) treatment). Presented data are the result of three separate experiments (n = 3/group) with each carried out in triplicate. All blot signals were normalized to total protein content. Following significant ANOVAs, Tukey’s pairwise comparison was used for post hoc comparisons. **B.** The bottom schematic depicts the possible route through which CTEP and rapamycin may influence autophagic functioning. The interaction of CTEP with the mGlur5 receptor is posited to reduce AKT, mTOR and p70S6 activity. This in turn, disinhibits ULK1 and subsequently catalyzes PI3K-dependent interactions with the Atg5-Atg12 complex. The Atg12–Atg5-Atg16 complex is then essential for autophagosome formation. Rapamycin ultimately would result in basically the same outcome as CTEP; however, rapamycin would interact downstream of mGlur5 at the level of mTOR

The levels of the autophagic associated proteins, Atg5 and Atg 12, were altered by the experimental treatments. Specifically, Atg5 was found to vary as a function of a (Vehicle vs CTEP vs Rapamycin) x (No Fibril vs Fibril) interaction (F(2,24) = 14.92, p < 0.05). Indeed, the exogenously applied α-syn PFFs reduced Atg5 levels below that of controls (p < 0.05) and the CTEP and rapamycin treatments then reversed this effect. There was also a significant main effect for the Vehicle/CTEP/Rapamycin treatment upon Atg12 levels F(2,24) = 18.01, p < 0.05. In this case, the CTEP and rapamycin treatments increased Atg12 levels, relative to vehicle treatment (p < 0.05; Fig. 4). Although there was no significant interaction, it was apparent that the impact of CTEP and rapamycin was confined to cells that also received the α-syn fibrils. Curiously, the A53T AV had no impact upon Atg5 or Atg12 levels, nor did CTEP or rapamycin affect these cells. Of course, there was also additional variability with the viral treatment.

## Discussion

We currently found that exogenously applied PFFs readily induced the accumulation of α-syn (detected using an aggregate conformation specific antibody) in SH-SY5Y human dopaminergic neurons and this effect was modestly augmented by additional adenoviral induced α-syn A53T over-expression. This is consistent with previous studies showing A53T to augment α-syn misfolding and aggregation, as well as facilitating the seeding between neurons [35, 41]. We also show that mGluR5 antagonism (using the negative allosteric regulator, CTEP) or mTOR inhibition (using rapamycin) reduced levels of α-syn in cultured human dopaminergic SH-SY5Y cells. Specifically, both CTEP and rapamycin treatment provoked a significant decrease in cellular levels of α-syn that occurred in response to exposure to the exogenous PFFs alone, or in the presence of A53T overexpression. Importantly, these SH-SY5Y neuronal cells had already been exposed to the α-syn PFF for six days prior to CTEP or rapamycin treatment. Hence, these treatments were likely increasing the clearance or breakdown of the already accumulated intracellular α-syn. It is also worth mentioning that we observed no obvious evidence of cellular death or toxicity in this study, which is consistent with what was previously reported after PFF treatment in SH-SY5Y cells within a 6-day time period [30]

The mTOR pathway is important for the autophagic removal of accumulated pathological proteins [16], raising the possibility that targeting this pathway might be useful for inducing the degradation of intracellular α-syn aggregates. Since mTOR activation normally has a tonic inhibitory influence over autophagy, rapamycin treatment or blocking upstream mGluR5 would be expected to result in a disinhibition of this process, thereby allowing for enhanced autophagic initiation. In fact, we did find that the CTEP and rapamycin reduced the activation of the mTOR pathway in the SH-SY5Y cells, while upregulating autophagic associated factors. Specifically, CTEP and rapamycin reduced the levels of phosphorylated mTOR relative to its total levels, as well as diminishing levels of the upstream and downstream pathway proteins, AKT and p70S6, respectively. Similarly, the alkaloid, Corynoxine B, was reported to induce an autophagic breakdown of α-syn that was associated with reductions in phospho-Akt, phospho-mTOR and phospho-p70S6 [9]. A recent paper likewise reported that the flavonoid, tricin, also provoked a presumably autophagic-dependent (reduction in AMPK, p70S6 and ATG7) degradation of α-syn [47]. Even more specific targeting of elements of the mTOR autophagic pathway, such as AKT [15], may also hold promise for therapeutic elimination of α-syn aggregates. Finally, it is also important that we found that the exogenous α-syn reduced levels of the autophagic proteins, Atg5 and Atg12, and that the CTEP and rapamycin treatments reversed this effect. The fact that activity of the Atg12–Atg5-Atg16 complex is essential for autophagosome formation is further support for an autophagic role in α-syn PFF clearance.

Given the tremendous variability between α-syn antibodies, it is important to note that the α-syn antibody we used (MJFR-14) was reported to be specific for aggregated forms of α-syn [11, 19, 33]. A recent comprehensive study that compared binding properties of multiple α-syn antibodies confirmed that MJFR-14 does indeed preferentially bind to fibrils and oligomers and not monomers at relatively low concentrations [21]. However, the antibody did show some weak binding to monomers at higher concentrations, but the authors do still state that MJFR-14 has high immunoreactivity with regards to aggregated α-syn forms [21]. It is noteworthy that the A53T AV driven α-syn labelling was clearly different than that induced by the PFFs (less punctate and more diffuse). It was also curious that CTEP and rapamycin did not affect this basal signal, but rather appeared to be selective for the PFF induced response. It could be that the autophagic processes induced by these pharmacological agents were more effective in reducing exogenous fibrillar forms of α-syn, than whatever form was induced by viral overexpression.

Besides intracellular degradation processes, it might also be therapeutically useful to target mechanisms that facilitate α-syn cell entry or spread. Accumulating evidence has suggested that α-syn enters and moves between cells by way of the transmembrane cellular prion protein, which is linked to the mGluR5 receptor [10, 13, 20, 25, 31, 45]. Moreover, oligomeric α-syn was found to promote the phosphorylation of the mGluR5 receptor resulting in synaptic and cognitive deficits [13]. The fact that mGlur5 inhibition reversed this effect [13], suggests that mGluR5 might be a useful target to impede α-syn cellular entry and/or spread. Importantly, the SH-SY5Y dopaminergic human derived neurons presently used have been demonstrated to indeed express the transmembrane cellular prion protein [26]. Yet, given that CTEP and rapamycin were presently given long after PFF exposure, they are unlikely to be acting to limit the a-syn cellular entry.

An alternate mechanism for action of CTEP and rapamycin is through the induction of α-syn ejection out of the SH-SY5Y neurons. It is possible that the present mTOR changes could be aligned with active ejection or passive efflux of α-syn owing to changes in cellular ion or other channels, possibly related to electrical polarization state. Indeed, mTOR has been proposed to be able to regulate voltage gated potassium channels [27], thereby influencing cellular excitability. Furthermore, we previously found that functional membrane depolarization by direct current stimulation reduced α-syn accumulation in PFF-treated SH-SY5Y cells [32]. This depolarizing treatment increased extracellular levels of α-syn but had no cytotoxic effects (Ross et al., 2020), suggesting the ejection of the accumulated protein. This also supports the possibility that electroconvulsive therapeutic applications that have proven efficacy in PD patients [40] might, at least in part, modulate α-syn aggregation.

Our data indicate that it may be feasible to eliminate α-syn aggregates by selectively targeting mGluR5 or mTOR pathways. This is in agreement with our previous findings linking mGluR5 activity with modulation of the mTOR pathway in other disease models [2, 3, 12]. In particular, the mGluR5 negative allosteric regulatory drug, CTEP, may provide a novel means of triggering α-syn degradation by engaging mTOR related autophagic mechanisms. Such allosteric acting compounds have favorable kinetic and safety profiles that might be particularly useful for promoting the breakdown or cellular ejection of potentially toxic proteinaceous aggregates that occur across a variety of neurodegenerative diseases.

## Data Availability

The datasets used and/or analyzed for the current study are available from the corresponding author upon reasonable request.

## Abbreviations

AMPA: α-Amino-3-hydroxy-5-methyl-4-isoxazolepropionic acid receptor (AMPA)
α-syn: α-Synuclein
CTEP: 2-Chloro-4-[2-[2,5-dimethyl-1-[4 (trifluoromethoxy)phenyl]imidazole 4yl]ethynyl] pyridine
mTOR: Mammalian target of rapamycin
PD: Parkinson’s disease
PFF: Preformed fibrils
RAP: Rapamycin
